# How the Signaling Crosstalk of B Cell Receptor (BCR) and Co-Receptors Regulates Antibody Class Switch Recombination: A New Perspective of Checkpoints of BCR Signaling

**DOI:** 10.3389/fimmu.2021.663443

**Published:** 2021-03-25

**Authors:** Zhangguo Chen, Jing H. Wang

**Affiliations:** ^1^ Department of Immunology and Microbiology, University of Colorado, Aurora, CO, United States; ^2^ Department of Medicine, Division of Hematology and Oncology, UPMC Hillman Cancer Center, University of Pittsburgh School of Medicine, Pittsburgh, PA, United States

**Keywords:** B cell receptor, class switch recombination, activation-induced deaminase, tumor necrosis factor receptor-associated factor-3, B cell homeostasis

## Abstract

Mature B cells express B cell antigen receptor (BCR), toll-like receptors (TLR) and TNF family receptors including CD40 and B-cell activating factor receptor (BAFFR). These receptors transduce cellular signals to govern the physiological and pathological processes in B cells including B cell development and differentiation, survival, proliferation, and antibody-mediated immune responses as well as autoimmune diseases and B cell lymphomagenesis. Effective antibody-mediated immune responses require class switch recombination (CSR), a somatic DNA recombination event occurring at the immunoglobulin heavy chain (*Igh*) gene locus. Mature B cells initially express IgM as their BCR, and CSR enables the B cells to switch from expressing IgM to expressing different classes of antibodies including IgG, IgA or IgE that exhibit distinct effector functions. Here, we briefly review recent findings about how the signaling crosstalk of the BCR with TLRs, CD40 and BAFFR regulates CSR, antibody-mediate immune responses, and B cell anergy.

## Introduction

**Figure 1:**
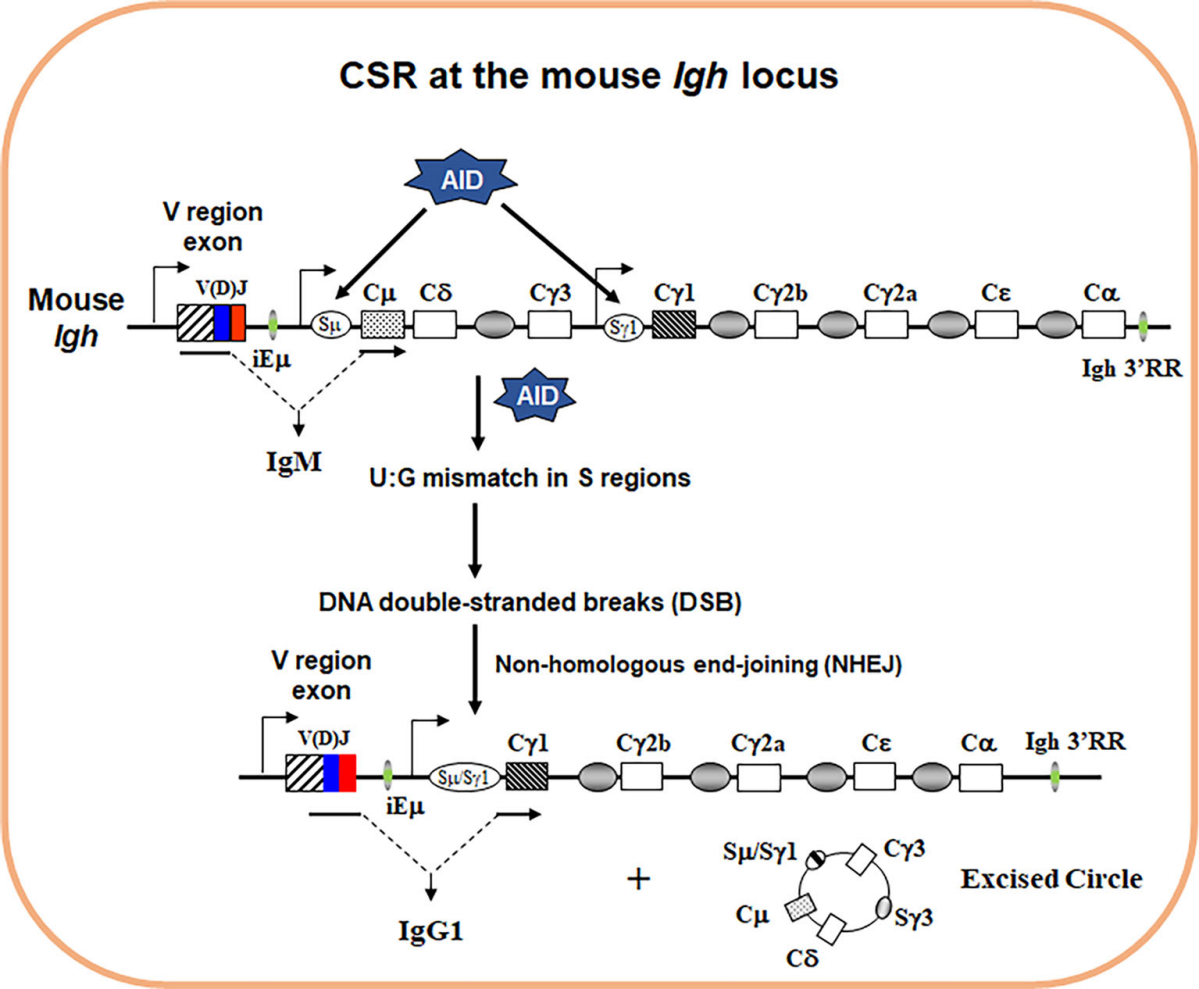
Schematics of IgH CSR. The genomic configuration of the rearranged *Igh* locus in mouse. Variable (V) region exon is located upstream, and eight different sets of C_H_ exons are located downstream. AID introduces point mutations into V region exon during somatic hypermutation (SHM) (not depicted). To initiate CSR, AID introduces U:G mismatches in the donor Sµ and the downstream acceptor Sγ1 regions that are subsequently converted into DNA double-stranded breaks (DSBs) by basic excision and mismatch repair pathways. Broken S regions are joined by non-homologous end-joining (NHEJ), whereas the intervening DNA is excised as a circle. Active transcription is essential for both SHM/CSR with promoters depicted for V region, Sµ and Sγ1 region (arrows). When CSR is completed, originally expressed Cµ exons are replaced by Cγ1 exons that are juxtaposed to the same V region exon. Therefore, naïve IgM^+^ B cells switch to activated IgG1^+^ B cells.

Antibody is also known as immunoglobulin (Ig), consisting of a heavy (IgH) and a light (IgL) chain. Each IgH molecule is composed of an assembled variable (V) region and a constant (C) region. Antigen contact is mediated by the V region, while the C region of IgH mediates effector functions of antibodies. Productive V(D)J recombination at the *Igh* locus assembles the V region exon that is located upstream of the Cµ IgH constant region exons, allowing generation of µ heavy chain mRNA and µ heavy chain protein. The µ heavy chains pair with IgL chains that are produced from a productively rearranged *Igl* locus to form IgM, which lead to generation of surface IgM^+^ B cells. IgM^+^ B cells migrate to secondary lymphoid organs such as spleen or lymph nodes, where upon encounter with antigens they are activated to undergo class switch recombination (CSR), a somatic DNA recombination/deletion process that replaces Cµ with a different set of IgH constant region exons (**Figure 1**).

In mice, there are 8 sets of C_H_ exons organized as 5’–VDJ–Cµ–Cδ–Cγ3–Cγ1–Cγ2b–Cγ2a–Cϵ–Cα–3’. CSR is a DNA rearrangement process that occurs to the 8 sets of C_H_ exons at the *Igh* locus (**Figure 1**). CSR allows the assembled V region to be expressed first with Cµ exons and then with one of the sets of downstream C_H_ exons (referred to as C_H_ genes), and enables production of different IgH classes (e.g., IgG, IgE, and IgA) encoded by corresponding C_H_ genes (e.g., Cγ, Cϵ, and Cα). The detailed molecular mechanisms of CSR have been extensively reviewed elsewhere (1–4). Briefly, to initiate CSR, B cells need to express a specific enzyme, activation-induced cytidine deaminase (AID) (5, 6). AID introduces DNA lesions to the evolutionarily conserved switch (S) regions preceding each set of C_H_ exons; subsequently, AID-induced DNA lesions are converted into double-stranded DNA breaks (DSBs) at the upstream donor Sµ region and one of the downstream acceptor S regions (7). DSBs at S regions are joined by non-homologous end-joining (NHEJ) pathway including classical and alternative NHEJ (8–11), which eventually leads to the switching of the C regions of antibody molecules. Of note, AID can potentially target all transcriptionally active genes and induces genome-wide instability that contributes to B cell lymphomagenesis (12, 13). Thus, AID poses a threat to the B cell genome and its expression has to be tightly regulated. Consequently, AID expression is only induced in activated B cells *via* integrated signals from the B cell antigen receptor (BCR) and other co-receptors (3).

Antibody CSR is essential for effective humoral immune responses. Mature naïve B cells express IgM as surface BCR or secrete IgM antibodies; however, effector functions of IgM are rather limited (3, 14, 15). CSR enables B cells to produce isotype-switched antibodies, such as IgG and IgA, that can combat infectious pathogens or neutralize toxins much more effectively than IgM. Consequently, more than 90% of current vaccines deliver protective effects *via* eliciting isotype-switched antibodies (16). On the other hand, defects in CSR lead to primary immunodeficiency diseases (PID) such as Hyper-IgM syndrome (HIGM) caused by genetic mutations in BCR or co-receptor signaling components (e.g., CD19 or CD40) (17, 18). In addition, HIGM can be caused by mutations in AID or uracil glycosylase that are essential enzymes to catalyze CSR (17, 18). PID patients suffer from recurrent infections with a shorter life expectancy (19–21). Hence, it is critical to better understand how the signaling crosstalk of BCR and co-receptors regulates antibody CSR.

## Can the BCR Induce CSR in the Absence of Co-stimulation?

Pathogen infection or antigen immunization activates multiple receptors on B cells including BCR, CD40, toll-like receptors (TLRs), B-cell activating factor receptor (BAFFR) and cytokine receptors (e.g., IL-4R) depending on different antigen characteristics. The prevailing view of CSR induction is that the BCR cannot induce CSR in the absence of co-stimulation, and the co-stimulatory signals are provided in the form of CD40 ligand (CD40L) expressed by activated T cells for T-cell dependent (TD) antigens, or TLR ligands directly expressed by pathogens or present in the adjuvants for T-cell independent (TI) antigens. Given that it is not practically feasible yet to pinpoint which and how individual receptor(s) induce CSR during *in vivo* immunization, *ex vivo* CSR models have been established and widely applied to study underlying mechanisms of CSR by culturing purified B cells in the presence of different stimuli and analyzing CSR level a few days after culture (2, 22).

It is well-known that engaging CD40 can induce CSR in the presence of proper cytokines such as IL-4 (2, 22). TLRs can also induce a low level of CSR in the presence of cytokines; moreover, TLRs synergize with the BCR to induce a robust level of CSR (23). In contrast, engaging the BCR cannot induce CSR in the presence of cytokines such as IL-4 (23, 24). The BCR recognizes antigen and activates multiple signaling pathways, including nuclear factor kappa B (NF-κB) and phosphatidylinositol 3-kinases (PI3K), to initiate antigen-specific humoral immune response. Hence, it is counterintuitive why the BCR cannot induce CSR in the presence of cytokines as CD40 does (3). Basically, why does the BCR need co-stimulation to induce CSR and what does co-stimulation do to enable the BCR to induce CSR?

**Figure 2:**
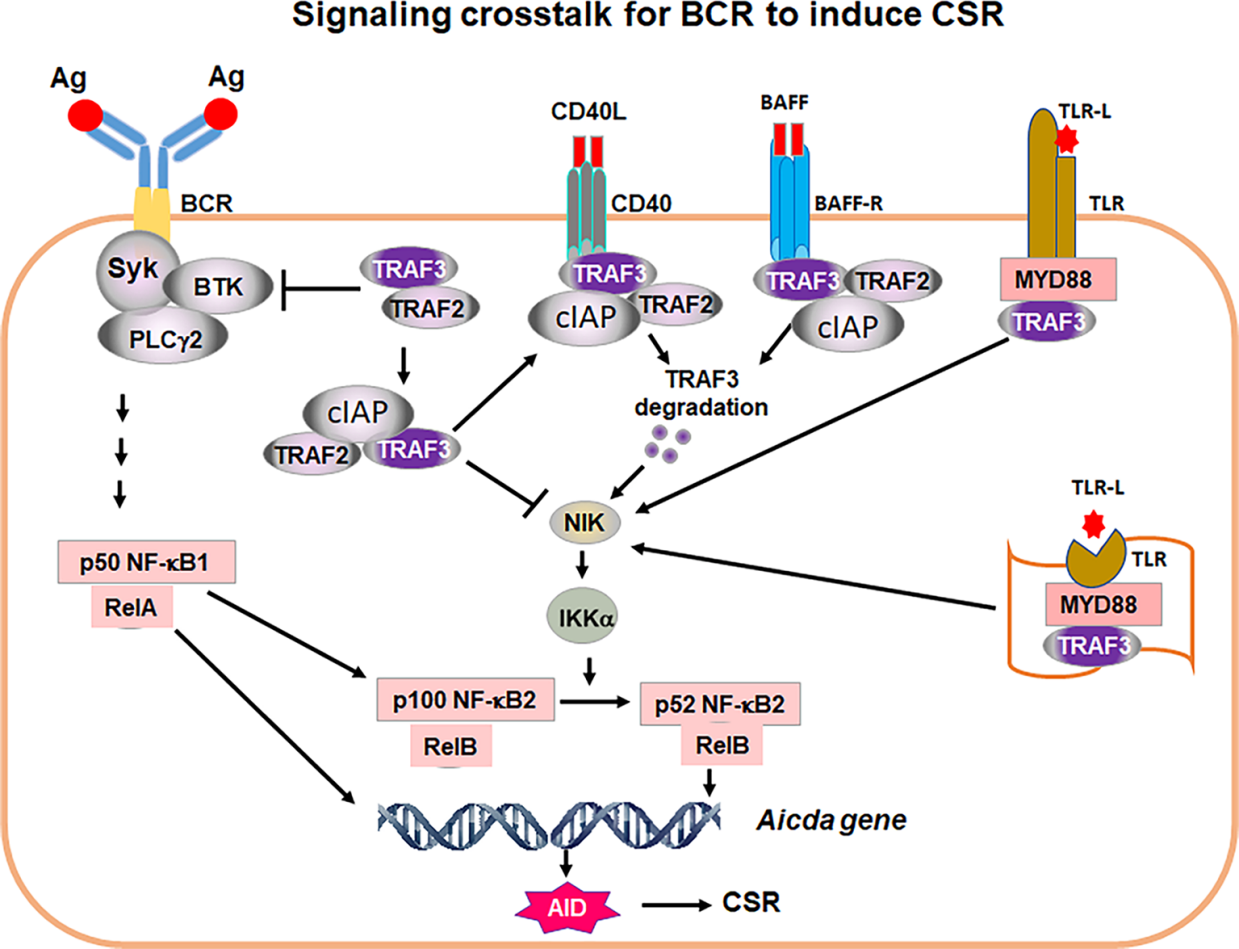
A proposed model of signaling crosstalk for the BCR to induce CSR. Ag stimulation of BCR activates proximal signaling elements, Syk, BTK and PLCγ2, leading to transcription factor NF-kB1 activation. NF-κB1 p50/RelA complex is required for AID transcription. NF-κB1 p50/RelA also induces NF-kB2 p100 transcription. TRAF2/3 restrict BCR proximal signaling strength. TRAF2 and TRAF3 also block NIK activity. Thus, Syk/BTK/PLCγ2 complex cannot signal to generate transcription factor NF-κB2 p52 that is required for AID expression. Removal of TRAF3 and/or TRAF2 leads to NIK accumulation, which activates IKKα pathway, resulting in NF-κB2 p100 being processed into active NF-κB2 p52. NF-κB2 p52/RelB complex and NF-κB1 p50/RelA together with additional factors initiate AID transcription. AID protein initiates CSR by targeting *Igh* locus. During humoral immune responses, CD40, BAFF-R as well as cell surface and intracellular TLRs are activated by corresponding ligands, CD40L, BAFF or TLR ligand (TLR-L), respectively. TRAF3/TRAF2 are recruited to cell membrane where TRAF3 is degraded by CD40 and BAFF-R signaling or sequestrated by TLRs. As a consequence, NIK and NF-κB2 complex can be activated. NF-κB2 activation allows the BCR to induce CSR. Thus, the critical function of co-stimulatory signals is to degrade or sequestrate TRAF3 to permit NF-κB2-dependent BCR-induced CSR essential for *in vivo* antibody responses. It is worthy of note that TRAF3 restricts Syk, BTK and PLCγ2 hyper-activation upon Ag stimulation that may be especially important for maintaining autoreactive B-cell anergy.

Our recent study has shed light on this decade long puzzle by revealing that there are negative regulatory mechanisms restricting the BCR’s ability to induce CSR (25). We identified two of such checkpoint molecules including TNF receptor associated factor 2/3 (TRAF2 and TRAF3) (**Figure 2**). When TRAF2 and/or TRAF3 are deleted in B cells, engaging the BCR can induce CSR in the absence of co-stimulation (25). These data demonstrate that the BCR has the ability to induce CSR; however, this ability is normally restrained by checkpoint molecules such as TRAF2 and TRAF3. Upon the deletion of these checkpoint molecules, the BCR’s need for co-stimulation to induce CSR can now be bypassed. We found that the BCR-induced CSR in the absence of TRAF3 requires NF-κB2 in a B cell-intrinsic manner (25). Mechanistically, TRAF3 restricted BCR signaling by preventing the processing of BCR-induced NF-κB2 precursor (p100) into active NF-κB2 (p52); consequently, TRAF3 deletion resulted in more active NF-κB2 (p52) upon anti-IgM/IL-4 stimulation (25). Of note, NF-κB2 activation is specifically required for the BCR signaling to induce CSR but not for CD40 or TLR4 (25, 26), suggesting that TRAF3 restricts NF-κB2 activation to specifically limit the BCR’s ability to induce CSR. Furthermore, we found that TRAF3 also inhibited BCR proximal signaling; as such, B-cell intrinsic deletion of TRAF3 led to elevated BCR proximal signaling strength, evidenced by increased phosphorylation of Bruton tyrosine kinase (BTK) and spleen tyrosine kinase (Syk) and enhanced calcium flux upon antigen or anti-IgM stimulation (25). While our recent study addressed how TRAF3 inhibits the BCR signaling, it remains unknown how TRAF2 regulates the BCR signaling intensity either singularly or cooperatively with TRAF3.

## How Do TRAF2 and TRAF3 Differentially Influence CSR and How Does the BCR Cooperate With Co-Receptors to Induce CSR?

Both TRAF2 and TRAF3 are adaptor molecules of TNF receptors (TNFRs) including CD40 and BAFFR and function to transmit signaling downstream of TNFRs (27). In resting B cells, TRAF2, TRAF3 and cellular inhibitor of apoptosis protein1/2 (cIAP1/2) form a complex to suppress NF-κB inducing kinase (NIK) activity by mediating NIK degradation (**Figure 2**). In activated B cells upon CD40 or BAFFR stimulation, TRAF3 can be degraded (25, 28, 29), thereby allowing NIK accumulation that eventually activates NF-κB2 (**Figure 2**). Although TRAF2 and TRAF3 both serve as adaptors of TNFRs and their individual knockout (KO) mice exhibited similar phenotypes (30–32), TRAF2 and TRAF3 play distinct roles in mediating CSR and *in vivo* antibody responses against TD or TI antigens.

With regard to CD40-induced CSR, we found that TRAF2 is required for CD40-induced AID expression and IgG1 CSR because TRAF2 plays an essential role in CD40-induced NF-κB1 activation (33). Consistently, B-cell intrinsic deletion of TRAF2 significantly impaired *in vivo* IgG antibody responses against TD antigens (33), given that TD antigen-induced IgG antibody responses need CD40/CD40L interaction. Contrary to the essential role of TRAF2 in CD40-induced CSR, TRAF3 is completely dispensable for CD40-induced AID expression and CSR (33). As such, B-cell intrinsic TRAF3 deletion did not affect IgG antibody responses against TD antigens *in vivo* (32, 33).

However, in the context of BCR-induced CSR, both TRAF2 and TRAF3 function as checkpoint molecules to prevent the BCR from inducing AID expression and CSR (25). This conclusion is supported by several important observations: (1) B cell-intrinsic TRAF2 deletion promotes the BCR-induced CSR *ex vivo*; (2) B cell-intrinsic TRAF3 deletion also promotes the BCR-induced CSR *ex vivo*, which occurs completely independent of any potential developmental effects; and (3) double deletion of TRAF2 and TRAF3 leads to a higher level of BCR-induced CSR than either single deletion does (25). In line with these observations, B-cell intrinsic deletion of TRAF2 or TRAF3 increased *in vivo* IgG antibody responses against TI antigens (33).

Of note, TI antigens can activate B cells in the absence of T cell help. Consistent with TRAF3’s role in restricting the BCR’s ability to induce CSR, we envision that increased IgG antibody responses against TI antigens are caused by elevated BCR signaling intensity *in vivo* upon TI antigen immunization in the absence of TRAF2 or TRAF3. However, this point remains to be determined; in addition, it remains unknown whether TRAF2/TRAF3 double KO mice will develop more robust IgG antibody responses against TI antigens. It is noteworthy that TRAF2 and TRAF3 play distinct roles in CD40-induced CSR and TD antigen-mediated responses, whereas they both function as checkpoints for BCR-induced CSR. Taken together, these studies highlight the complexity and fine-tuning potential of antibody-mediated immune responses that may have important implications for vaccine design of different types of antigens, such as TD vs. TI antigens.

If the BCR has the ability to induce CSR, why do B cells need co-stimulation and what does co-stimulation provide in the context of CSR induction? We suggest that CD40 aids BCR-induced CSR *in vivo* by inducing TRAF3 degradation (**Figure 2**), which is supported by our *ex vivo* studies showing that anti-CD40/IL-4 stimulation caused TRAF3 degradation in B cells (25). Subsequently, transient degradation of TRAF3 will allow NF-κB2 activation, AID expression and CSR induction. Once CD40 co-stimulation ceases, TRAF3 expression would resume and CSR would be terminated. However, this notion still needs to be tested in an *in vivo* setting. Regarding the role of BAFFR in CSR induction, our recent studies also suggest that BAFFR’s function is to degrade TRAF3, thus permitting the BCR to induce CSR (25) (**Figure 2**), although this point still needs to be confirmed experimentally. Nevertheless, this idea is supported by the observations that BAFF/IL-4 cannot induce a robust level of CSR, whereas BAFF/IL-4/anti-IgM induced a much higher level of IgG1 CSR that is not significantly enhanced in TRAF3 conditional KO B cells (25).

TLRs have been shown to synergize with the BCR to induce CSR by enhancing NF-κB2 activation, and such synergistic effects depend on a regulatory subunit of PI3Ks, p85 (23). However, the catalytic subunits of PI3Ks inhibit AID expression and CSR induced by CD40 (34), TLR4 (24) and BCR. Thus, the precise mechanisms remain elusive about how TLRs and the BCR synergize to enhance NF-κB2 activation to promote CSR. TLRs can bind TRAF3 *via* their adaptors myeloid differentiation primary response protein (MYD88) and TIR-domain-containing adapter-inducing interferon-β (TRIF). In contrast, TLRs do not induce TRAF3 degradation like CD40 does, because TRAF3 is required for TLR-induced cytokine production (35). Hence, we suggest that TLRs may sequester TRAF3 *via* adaptors MYD88 and/or TRIF, thereby releasing NIK that eventually activates NF-κB2 (p52) to induce AID expression and CSR (**Figure 2**).

## What Are the Consequences of BCR Checkpoint Removal?

When TRAF3 is deleted specifically in B cells *via* CD19Cre (B-TRAF3-KO), mice develop autoimmune manifestations including splenomegaly, lymphocyte infiltration in liver and immune-complex deposition in kidney at the age of 9-12 months (32). B-TRAF2-KO mice exhibit similar phenotypes to B-TRAF3-KO mice in B cell development and survival as well as lymph organ homeostasis (30–32). However, it remains incompletely understood how TRAF3 deficiency leads to autoimmune manifestations. Previous studies showed that B cell hyperplasia in B-TRAF3-KO mice was independent of BAFF-BAFFR signaling by using TACI-Ig, a soluble fusion protein that blocks both BAFF and APRIL from binding to their receptors (32). In addition, another study showed that the expansion of marginal zone (MZ) B cells in B-TRAF2-KO mice was independent of BAFF (30), suggesting that B-TRAF2-KO phenotypes were also independent of BAFF receptor signaling.

Based on our data (25), we suggest that the phenotypes of increased B cell number, splenomegaly, and autoimmune manifestations in B-TRAF3-KO mice depend on BCR signaling. We found that TRAF3-deficiency-mediated lymphoid organ disorders and autoimmune manifestations were rectified by attenuating BCR proximal signaling strength using a BTK inhibitor, Ibrutinib (25). Importantly, autoimmune phenotypes were completely rescued in B-TRAF3-KO mice by introducing an antigen-specific BCR recognizing hen egg lysozyme (HEL) (25). Given that B-TRAF3-KO mice do not express HEL antigens, these HEL-specific B cells cannot receive stimulatory signals from their BCR. We infer that introducing a non-autoreactive BCR abrogates the abnormal expansion of B cells and reduces the severity of autoimmunity. Furthermore, our data showed that NF-κB2 is required for the expansion of B cells, especially MZ B cells, and splenomegaly in B-TRAF3-KO mice (25). TRAF3 restricted BCR signaling by preventing the processing of BCR-induced NF-κB2 precursor (p100) into active NF-κB2 (p52) upon anti-IgM/IL-4 stimulation (25). Taken together, these data suggest that the phenotypes of B-TRAF3-KO mice largely attribute to dysregulated BCR signaling pathway.

Anergy is an important mechanism to maintain B cell tolerance *via* unresponsiveness of the BCR to antigen stimulation (36). Anergic autoreactive B cells express a low level of surface IgM that does not induce calcium flux when stimulated with specific antigens or agonist anti-IgM (37, 38). We employed a bone marrow chimera system to establish an anergic autoreactive model by transferring B cells with HEL-specific BCR into ML5 transgenic mice that constitutively express HEL antigens, and showed that B cell anergy was well maintained in this model (25), consistent with prior studies (37, 39, 40). In contrast, we found that autoreactive B cell anergy was broken by TRAF3-deficiency in HEL-specific B cells evidenced by enhanced calcium flux upon HEL antigen stimulation (25). If anergy is properly maintained, secreted anti-HEL IgM production should be reduced, and surface IgM expression downregulated, a classic anergy phenotype, upon HEL stimulation. However, TRAF3-deficiency enables HEL-specific B cells to produce a high level of anti-HEL IgM and fail to downregulate surface IgM expression (25). We think that TRAF3-deficiency breaks B cell anergy possibly *via* elevating BCR proximal signaling strength, which is supported by our findings that TRAF3 deletion enhances BCR-induced phosphorylation of Syk and BTK as well as phospholipase Cγ2-dependent calcium flux (25); however, it remains to be addressed how TRAF3 restricts BCR proximal signaling strength.

## Discussion

Our recent studies discover novel checkpoint molecules, including TRAF2 and TRAF3, that were not appreciated previously. We found that these checkpoint molecules function to restrict the ability of the BCR to induce AID expression and CSR. However, additional questions remain to be addressed. Deletion of TRAF2 or TRAF3 in B cells enhances BCR-induced calcium flux (25), an early functional output of the BCR signaling. What mechanisms are employed by TRAF2 and TRAF3 to restrict BCR signaling intensity? Are there other signaling components of the BCR and co-receptor pathways that can also function as checkpoints and similarly affect the ability of the BCR to induce aberrant AID expression? How do TRAF2 and TRAF3 cooperate to restrain BCR-induced CSR since double deficiency of TRAF2 and TRAF3 does not further increase AID expression but significantly enhances CSR level compared to either single deficiency? Does TRAF2 deficiency break autoreactive B cell anergy as TRAF3 does? Addressing these questions may allow us to develop new strategies to rescue defective antibody responses in CD40-deficient mouse model or human PID patients, and to better treat autoimmunity or B cell lymphoma by modulating BCR signaling pathways.

Our data show that B cells from B-TRAF3-KO mice proliferate more robustly than control B cells upon BCR engagement (25). One unique characteristics of germinal center (GC) B cells is their hyperproliferative capacity in the dark zone of GCs where somatic hypermutation (SHM) is thought to occur (41). Prior studies also showed that B-TRAF3-KO mice developed spontaneous GC formation (32). Thus, we speculate that TRAF2 and TRAF3 may also play a critical role in regulating SHM and antibody affinity maturation, which is the outcome of GC reaction.

Taken together, our studies present a new concept that may better explain how signaling components of the BCR and co-receptor pathways assure robust humoral immune responses while simultaneously preserve B cell homeostasis and prevent malignancy by fine-tuning the BCR signaling intensity. We propose that when BCR signaling intensity is increased to a level sufficient to induce AID expression and CSR, it may disrupt autoreactive B cell tolerance and perturb B cell homeostasis. Moreover, AID can induce DSBs and mutations in Ig and non-Ig genes that may initiate B cell genomic instability and lymphomagenesis. Thus, it may be critical to restrain the BCR from inducing AID expression in the absence of co-stimulation because this could serve as a protective mechanism that prevents overstimulated self-reactive B cells from turning cancerous.

## Author Contributions

ZC and JW wrote the manuscript. All authors contributed to the article and approved the submitted version.

## Funding

This work was supported by UPMC Hillman Cancer Center startup funds to JHW, R21-CA184707, R21-AI110777, R01-CA166325, R21-AI133110, R01-CA229174 and R01-CA249940 to JHW, and a fund from American Cancer Society (ACS IRG #16-184-56) to ZC. The sponsors or funders have no role in the preparation, review, or approval of the manuscript.

## Conflict of Interest

The authors declare that the research was conducted in the absence of any commercial or financial relationships that could be construed as a potential conflict of interest.
